# Cancer Cachexia and Dysregulated Phosphate Metabolism: Insights from Mutant p53 and Mutant Klotho Mouse Models

**DOI:** 10.3390/metabo12121284

**Published:** 2022-12-17

**Authors:** Ronald B. Brown

**Affiliations:** School of Public Health Sciences, University of Waterloo, Waterloo, ON N2L 3G1, Canada; r26brown@uwaterloo.ca

**Keywords:** cancer cachexia, phosphate toxicity, dysregulated phosphate metabolism, klotho, mutant p53 mice, mutant klotho mice, muscle wasting, aging, bone disorders

## Abstract

The present perspective article proposes that cachexia, muscle wasting in cancer, is mediated by dysregulated phosphate metabolism and phosphate toxicity that can damage tissues in most major organ systems. A diet high in phosphorus fed to mice deficient in klotho, a cofactor that regulates phosphate metabolism, accelerates aging, sarcopenia, general organ atrophy, kyphosis, and osteoporosis. Similar effects are seen in phenotypes of mutant p53 mice that overexpress the p53 tumor suppressor gene. Although mutant p53 mice do not develop tumors compared to wild-type mice, mutant p53 mice have shorter mean lifespans. Furthermore, tumorigenesis is associated with the sequestration of excessive inorganic phosphate, and dangerous levels of phosphate are released into circulation during tumor lysis syndrome. In total, this evidence implies that tumorigenesis may be a compensatory mechanism that provides protective effects against systemic exposure to dysregulated phosphate metabolism and phosphate toxicity related to cachexia in cancer. Moreover, the hypothetical protection against phosphate toxicity afforded by tumorigenesis also provides an alternate explanation for putative tumor evasion of the immune system. Insights proposed in this perspective paper provide new directions for further research, with potential to develop novel interventions and clinical applications that modify dietary phosphate intake to reduce cachexia in cancer patients.

## 1. Introduction

The metabolism of the essential dietary mineral phosphorus in the form of inorganic phosphate (Pi) is regulated in the human body by a sensitive network of hormones from bone, kidneys, intestines, and parathyroid glands [1]. Fibroblast growth factor 23 (FGF23) from bone, and parathyroid hormone (PTH) from the parathyroid glands, downregulate high Pi serum levels by decreasing renal Pi reabsorption and increasing phosphaturia. Conversely, the kidneys produce bioactive vitamin D (calcitriol), which increases Pi intestinal absorption and upregulates Pi serum levels. Overburdening this Pi regulatory network may produce phosphate toxicity, a condition in which high amounts of dysregulated phosphate accumulate in the body and damage most major organ systems. In animal studies, high dietary phosphate intake is associated with stimulated cell signaling through the phosphoinositide 3-kinase/AKT/mTOR pathway, leading to cancer growth as excessive Pi is sequestered into cells and incorporated into the sugar–phosphate backbone of nucleic acids during tumorigenesis [2].

A recent analysis of data from the U.S. National Health and Nutrition Survey (NHANES) found that cancer was inversely associated with serum levels of klotho, a transmembrane protein produced in kidneys and the brain that functions as a cofactor with FGF23 to downregulate serum Pi [3]. The forced expression of klotho in laboratory samples of cancer cells reduced cancer cell proliferation, while klotho silencing increased cancer cell proliferation [4], suggesting that klotho acts as a tumor suppressor in vitro. However, more research is needed to determine whether klotho’s association with tumor suppression is mediated by klotho’s role in downregulating Pi concentrations in cancer cells in vivo. Furthermore, reduced klotho levels in laboratory mice are associated with premature aging [5]. Of relevance, phosphate toxicity accelerates aging effects in laboratory mice [6], inferring that the downregulation of dysregulated phosphate by klotho may also mediate the association of klotho with “putative age-suppressing” properties [7]. In both cancer and aging, the reduction in phosphate toxicity potentially mediates klotho as both a tumor-suppressing agent and as an age-suppressing agent.

Muscle wasting in aging—sarcopenia—shares common pathways with muscle wasting in cancer—cachexia—and “more research should be devoted to the understanding of muscle wasting mediators, both in cancer and ageing” [8]. “For the practicing oncologist, early identification and management of cachexia is critical” [9]. Cachexia occurs in up to 80% of advanced cancer patients and directly causes 30% of cancer deaths [10], but effective treatments for cancer cachexia are still undergoing development. For example, a systematic review of pharmacotherapies for cachexia found promising benefits in clinical trials for anamorelin, a selective ghrelin receptor agonist [11]. Nevertheless, efforts to treat cancer cachexia with medical or nutritional interventions have failed in the past, and new multimodality approaches are needed to target “mechanisms contributing to cachexia” [12].

Muscle mass loss is also associated with early aging in a mutant mouse model of p53, a tumor suppressor [13], but a mechanism has not been proposed to explain how tumor suppression increases muscle loss. The present perspective paper investigates this issue by synthesizing evidence from models of mutant p53 mice and mutant klotho mice, providing novel insights into the relationship between muscle loss, phosphate toxicity, and cancer. Rather than “cancer-induced muscle wasting” [14], evidence suggests that phosphate toxicity independently induces both cancer and muscle wasting. Furthermore, by sequestering dysregulated phosphate, tumors hypothetically provide protection against tissue and organ damage from systemic exposure to circulating levels of excessive and toxic inorganic phosphate. Insights from the mechanisms proposed in this paper have potential for development into interventions that modify dietary phosphate intake to reduce and prevent phosphate toxicity and reduce cachexia in cancer patients. Such interventions may also assist in halting tumorigenesis and possibly regress tumors.

## 2. Method

A grounded theory method was used in this paper to rigorously and objectively review the research literature [15]. Research findings were retrieved relevant to cancer cachexia, muscle loss, and phosphate toxicity. Evidence from mutant mouse models, klotho knockout mice and p53 mutant mice, was selected for comparative analysis, and results were classified into pathophysiological mechanisms and concepts. Concepts and mechanisms were synthesized into epidemiological and etiological relationships as causative, associative, and mediating factors, until a cohesive theory explaining the cause of cancer cachexia was formed. More detailed information describing the method used in this paper is contained in the author’s work, Breakthrough Knowledge Synthesis in the Age of Google [16].

## 3. Klotho Knockout Mice and p53 Mutant Mice

### 3.1. Klotho Knockout Mice

The mutated klotho gene and its effect on aging was introduced in a mouse model by Kuro-o et al. in 1997 [5]. In 2010, Ohnishi and Razzaque [6] used a klotho knockout mouse model with an inactivated klotho gene to investigate the role of dietary phosphate feeding and phosphate toxicity in accelerating aging effects. Compared to wild-type mice, klotho mutant mice had higher serum phosphate levels associated with increased expression of the protein for sodium–phosphate cotransporters (NaPi2a) within the kidney nephrons. The results of experiments that altered genetic and dietary factors in klotho knockout mice were summarized by researchers as follows:

“Klotho knockout mice (*klotho^−/−^*) have a short life span and show numerous physical, biochemical, and morphological features consistent with premature aging, including kyphosis, uncoordinated movement, hypogonadism, infertility, severe skeletal muscle wasting, emphysema, and osteopenia, as well as generalized atrophy of the skin, intestine, thymus, and spleen”.

[6]

**Figure 1:**
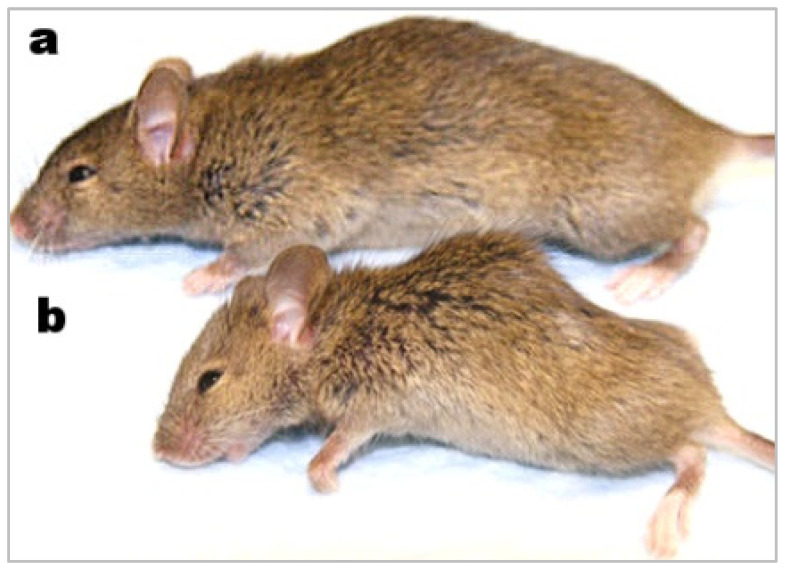
Wild-type mouse (**a**) and smaller-sized klotho mutant mouse with kyphosis (**b**).

All the above features in klotho knockout mice are associated with phosphate toxicity from feeding high dietary phosphate, as demonstrated by the researchers through genetic manipulation of sodium–phosphate cotransporters. Figure 1, from a study of wound healing in a klotho mutant mouse model, compares a wild-type mouse (a) to a smaller-sized klotho mutant mouse with kyphosis (b) [17].

### 3.2. p53 Mutant Mice

The activation of the p53 protein is linked to cellular senescence, “an irreversible process of growth arrest occurring in response to cellular aging” which also serves as a tumor suppression mechanism [18]. In 2002, Tyner et al. reported generating a p53 mouse with a genetically altered ‘m’ allele that augments tumor suppression (*p53^+/m^*) [13]. Comparisons with wild-type mice (*p53^+/+^*) include the following findings:

“Skinned, older *p53^+/m^* mice exhibit clear reductions in body mass, adipose tissue deposition, muscle mass and a pronounced lordokyphosis”.

[13]

**Figure 2:**
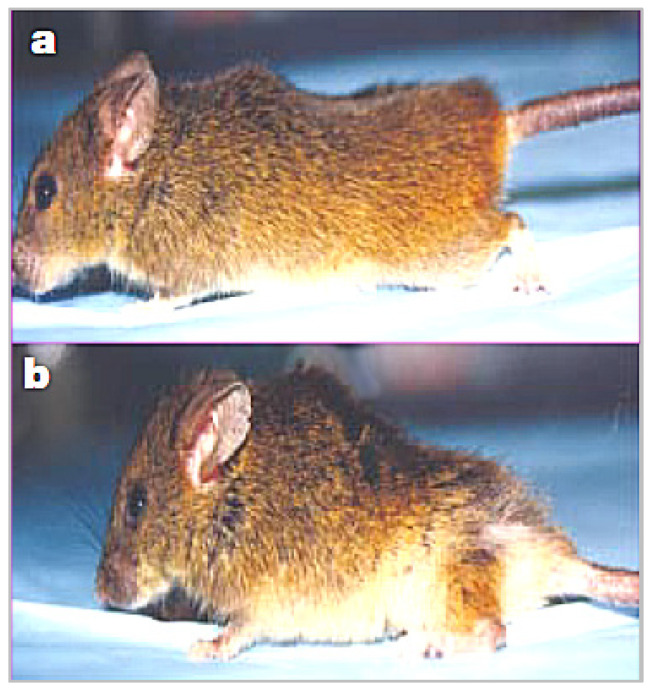
Wild-type mouse (**a**) compared to (**b**), smaller-sized p53 mutant mouse with kyphosis.

The mean mass of quadricep muscles in mutant p53 mice was 2.5-fold lower than in wild-type mice. Although over 45% of wild-type mice developed tumors, the mutant p53 mice “were highly resistant to spontaneous tumours” [13]. Nevertheless, contrary to expectations, mutant p53 mice had a shorter mean lifespan of 96 weeks compared to 118 weeks in wild-type mice. Additionally, osteoporosis and atrophy of skin, spleen, and other organs occurred in mutant p53 mice. Figure 2 compares a wild-type mouse from the study (a) with a smaller-sized p53 mutant mouse with kyphosis (b).

## 4. Discussion

To the best of the author’s knowledge, the present study is the first to compare mechanisms linking klotho and p53 to cancer cachexia. Neither of the two mutant mouse models selected for comparative analysis in this study were originally intended as specific models for cancer cachexia, focusing instead on aging effects. Nevertheless, severe muscle loss and organ atrophy are effects common to both aging and cancer cachexia, and a comparison of the model results offer clues in the etiologies shared by aging and cancer cachexia. Furthermore, the congenital genetic mutations in these experimental models were not intended to represent clinical cachexia incidence as it develops in real-life cancer patients. The genetic alterations, however, provide experimental variables necessary in the research design for comparison with control variables of the wild-type mice. Overall, a comparison of the phenotypes of these experimental models provides novel insights that are relevant to the interpretation of morphological changes and pathophysiological mechanisms in clinical cachexia of cancer patients.

The similarity of phenotype features in p53 mutant mice with those of klotho mutant mice is striking, inferring a similar pathophysiological mechanism involving phosphate toxicity from dysregulated phosphate metabolism related to high dietary phosphate intake. “Severe skeletal muscle wasting” in mutant klotho mice and loss of body mass and muscle mass in mutant p53 mice is especially relevant to the association of cancer cachexia with phosphate toxicity. A potential pathophysiological mechanism associating muscle-mass loss with phosphate toxicity is suggested in more recent research, linking phosphate toxicity to mitochondrial dysfunction and cell death from opening of the mitochondrial permeability transition pore [19]. Moreover, skeletal changes in both mutant p53 mice and mutant klotho mice, including kyphosis, osteopenia, and osteoporosis, are consistent with disorders of calcium and phosphorus metabolism [20]. Relevant to findings suggesting dysregulated phosphate metabolism in mutant klotho mice and mutant p53 mice, a model for cancer research by Schipper et al. [21] proposed that cancer develops from dysregulated metabolism, and that this dysregulation may be reversable.

Importantly, the unexpected finding of a shorter mean lifespan with tumor suppression in the mutant p53 mice suggests that tumorigenesis may provide protection against systemic phosphate toxicity by sequestering and lowering excessive concentrations of circulating Pi. Evidence in support of this implication includes tumor lysis syndrome, an emergency condition in which large amounts of Pi and other electrolytes stored in tumors are released into circulation upon lysis of tumor cells during treatment:

“These electrolyte and metabolic disturbances can progress to clinical toxic effects, including renal insufficiency, cardiac arrhythmias, seizures, and death due to multiorgan failure”.

[22]

**Figure 3:**
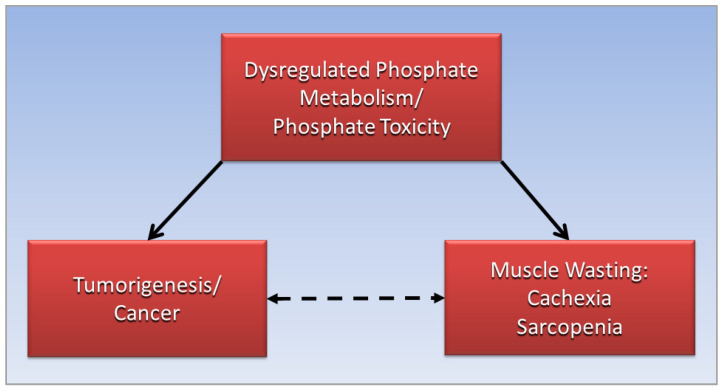
Dysregulated phosphate metabolism and phosphate toxicity mediate the association of cancer with cachexia.

Furthermore, a hypothetical protective mechanism provided by tumorigenesis against damaging effects of systemic phosphate toxicity could explain the putative “immune evasion” of tumors that avoids destruction by the immune system [23]. Alternatively, the present paper proposes a cooperative immune response and accommodation of tumorigenesis as a compensatory response to defend against systemic phosphate toxicity. Further research is needed to investigate novel insights in the present paper. Figure 3 graphically summarizes relationships proposing that the association of cancer with cachexia, the dotted arrow, is mediated by dysregulated phosphate metabolism and phosphate toxicity, which independently cause cancer and cachexia, represented by solid arrows.

## 5. Conclusions

More effective treatments are needed for cancer cachexia. This paper proposes that dysregulated phosphate metabolism and phosphate toxicity mediate muscle-wasting cachexia in cancer patients. Features of mutant p53 mice and mutant klotho mice are strikingly similar, including muscle-mass loss and bone disorders, and these features have been linked to dysregulated phosphate from high dietary phosphate intake. Additionally, a decreased lifespan in mutant p53 mice with tumor suppression compared to wild-type mice with over 45% of tumor incidence suggests that tumorigenesis may provide a protective effect against systemic phosphate toxicity by sequestering and lowering high concentrations of phosphate in circulation. Hypothetical protection provided by tumorigenesis challenges the premise of tumor evasion of the immune system. The proposals in this paper are consistent with a model of cancer research, suggesting that cancer develops from dysregulated metabolism, and that this dysregulation may be reversable. Insights provided in this paper offer new directions that warrant further investigation, with potential for development of novel interventions and clinical applications that modify dietary phosphate intake to reduce cachexia in cancer patients.

## References

[1] Brown R.B., Razzaque M.S., Singh A.K., Williams G.H. (2018). Chapter 31—Endocrine Regulation of Phosphate Homeostasis. Textbook of Nephro-Endocrinology.

[2] Brown R.B., Razzaque M.S. (2018). Phosphate toxicity and tumorigenesis. Biochim. Biophys. Acta (BBA)—Rev. Cancer.

[3] Qiao Y., Liu F., Peng Y., Wang P., Ma B., Li L., Si C., Wang X., Zhang M., Song F. (2022). Association of serum Klotho levels with cancer and cancer mortality: Evidence from National Health and Nutrition Examination Survey. Cancer Med..

[4] Wolf I., Levanon-Cohen S., Bose S., Ligumsky H., Sredni B., Kanety H., Kuro-o M., Karlan B., Kaufman B., Koeffler H.P. (2008). Klotho: A tumor suppressor and a modulator of the IGF-1 and FGF pathways in human breast cancer. Oncogene.

[5] Kuro-o M., Matsumura Y., Aizawa H., Kawaguchi H., Suga T., Utsugi T., Ohyama Y., Kurabayashi M., Kaname T., Kume E. (1997). Mutation of the mouse klotho gene leads to a syndrome resembling ageing. Nature.

[6] Ohnishi M., Razzaque M.S. (2010). Dietary and genetic evidence for phosphate toxicity accelerating mammalian aging. FASEB J..

[7] Kuro-o M. (2011). Klotho and the aging process. Korean J. Intern. Med..

[8] Argilés J.M., Busquets S., Felipe A., López-Soriano F.J. (2006). Muscle wasting in cancer and ageing: Cachexia versus sarcopenia. Adv. Gerontol..

[9] Bruggeman A.R., Kamal A.H., LeBlanc T.W., Ma J.D., Baracos V.E., Roeland E.J. (2016). Cancer Cachexia: Beyond Weight Loss. J. Oncol. Pract..

[10] National Cancer Institute Treating Cancer Cachexia: Progress Looks Possible. https://www.cancer.gov/about-cancer/treatment/research/cachexia.

[11] Advani S.M., Advani P.G., VonVille H.M., Jafri S.H. (2018). Pharmacological management of cachexia in adult cancer patients: A systematic review of clinical trials. BMC Cancer.

[12] Del Fabbro E. (2010). More is better: A multimodality approach to cancer cachexia. Oncologist.

[13] Tyner S.D., Venkatachalam S., Choi J., Jones S., Ghebranious N., Igelmann H., Lu X., Soron G., Cooper B., Brayton C. (2002). p53 mutant mice that display early ageing-associated phenotypes. Nature.

[14] Aversa Z., Costelli P., Muscaritoli M. (2017). Cancer-induced muscle wasting: Latest findings in prevention and treatment. Ther. Adv. Med. Oncol..

[15] Wolfswinkel J.F., Furtmueller E., Wilderom C.P.M. (2013). Using grounded theory as a method for rigorously reviewing literature. Eur. J. Inf. Syst..

[16] Brown R.B. (2020). Breakthrough knowledge synthesis in the Age of Google. Philosophies.

[17] Yamashita K., Yotsuyanagi T., Yamauchi M., Young D.M. (2014). Klotho Mice: A Novel Wound Model of Aged Skin. Plast. Reconstr. Surg. Glob. Open.

[18] Mijit M., Caracciolo V., Melillo A., Amicarelli F., Giordano A. (2020). Role of p53 in the Regulation of Cellular Senescence. Biomolecules.

[19] Nguyen T. (2016). Mitochondrial reactive oxygen species, endoplasmic reticulum stress and phosphate toxicity in impairment of pancreatic cells. TTU Rev..

[20] Sun M., Wu X., Yu Y., Wang L., Xie D., Zhang Z., Chen L., Lu A., Zhang G., Li F. (2020). Disorders of Calcium and Phosphorus Metabolism and the Proteomics/Metabolomics-Based Research. Front. Cell Dev. Biol..

[21] Schipper H., Turley E.A., Baum M. (1996). A new biological framework for cancer research. Lancet.

[22] Howard S.C., Jones D.P., Pui C.H. (2011). The tumor lysis syndrome. N. Engl. J. Med..

[23] Kim S.K., Cho S.W. (2022). The Evasion Mechanisms of Cancer Immunity and Drug Intervention in the Tumor Microenvironment. Front. Pharmacol..

